# Consent-driven, semi-automated data collection during birth and newborn resuscitation: Insights from the NewbornTime study

**DOI:** 10.1371/journal.pdig.0000730

**Published:** 2025-09-08

**Authors:** Sara Brunner, Anders Johannessen, Jorge García-Torres, Ferhat Özgur Catak, Øyvind Meinich-Bache, Siren Rettedal, Kjersti Engan

**Affiliations:** 1 Laerdal Medical AS, Stavanger, Norway; 2 Department of Electrical Engineering and Computer Science, University of Stavanger, Stavanger, Norway; 3 bitUnitor AS, Stavanger, Norway; 4 Faculty of Health Sciences, University of Stavanger, Stavanger, Norway; 5 Department of Pediatrics, Stavanger University Hospital, Stavanger, Norway; Fundación Progreso y Salud: Junta de Andalucia Consejeria de Salud y Familias Fundacion Progreso y Salud, SPAIN

## Abstract

Accurate observations at birth and during newborn resuscitation are fundamental for quality improvement initiatives and research. However, manual data collection methods often lack consistency and objectivity, are not scalable, and may raise privacy concerns. The NewbornTime project aims to develop an AI system that generates accurate timelines from birth and newborn resuscitation events by automated video recording and processing, providing a source of objective and consistent data. This work aims to describe the implementation of the data collection system that is necessary to support the project’s purpose. Videos were recorded using thermal sensors in labor rooms and thermal and visible light cameras in resuscitation rooms. Consent from mothers was obtained before birth, and healthcare providers were given the option to delete videos by opting out on a case-by-case basis. The video collection process was designed to minimize interference with ongoing treatment and not impose unnecessary burden on healthcare providers. At Stavanger University Hospital, 1012 thermal videos of birth and 274 visible light videos of newborn stabilization and resuscitation have been collected during the data collection period from November 2021 to June 2025. The utilization of automated data collection and AI video processing around birth may allow for consistent and enhanced documentation, quality improvement initiatives, and research opportunities on sequence, timing and duration of treatment activities during acute events, with less effort needed for capturing data and improved privacy for participants.

## Author summary

In our study, we address the need for accurate and consistent data collection during birth and newborn resuscitation. Traditional manual data collection methods often fall short in terms of objectivity, consistency, scalability, and privacy. To overcome these challenges, we have developed a consent-driven, semi-automated data collection system with the aim of developing AI-based systems that uses video recordings to create precise timelines of events occurring at birth and during newborn resuscitation. This method leverages thermal and visible light cameras to capture footage, ensuring minimal interference with clinical practices and respecting the privacy of participants. This work describes the detailed setup of the data collection and sets it in perspective to existing solutions used in clinical practice and research. From November 2021 to June 2025, we collected data at Stavanger University Hospital, including 1012 thermal videos of births and 274 visible light videos of newborn stabilization or resuscitation. This data will support the future development of AI algorithms aimed at enhancing the objectivity and consistency of data collection. Ultimately objective data can be used for individual learning or, on a big scale, understanding adherence to guidelines and effect of treatment, when combined with outcome data.

## Introduction

Data collection during birth and newborn resuscitation presents challenges. Manual observations in real time and annotation of video observations may have issues with objectivity, accuracy, privacy and scalability [1–4]. Accurate and high-quality data is needed for documentation, research, quality improvement initiatives and for learning purposes. Recently, Bettinger et al. [5] addressed the importance of making “every birth a learning event” to improve the competence of healthcare providers, as they seldom encounter newborn resuscitation in their clinical practice.

In their review, Avila-Alvarez et al. [1] concluded that the current quality of documentation in newborn resuscitation is unsatisfactory, highlighting the need for consensus guidelines and innovative solutions. While retrospective documentation may be less accurate than video recordings [2], real-time documentation has shown promising results in capturing interventions during newborn resuscitation [3].

Video recording of newborn stabilization treatment for quality improvement purposes is done in multiple sites around the world but is still far from scalable to routine practice in most places [6–8]. Continuous video recording and button-triggered recording are commonly used methods for capturing real-life emergency team footage for auditing [2]. We are aware of one hospital in the Netherlands that considers newborn treatment videos as standard care and stores them in the patient records [6], but most hospitals are deleting the videos after some days or weeks [8–10].

The NewbornTime project [11], registered in ISRCTN Registry [12], is a collaborative project between University of Stavanger (UiS), Stavanger University Hospital (SUH), bitUnitor AS and Laerdal Medical AS that aims to improve data collection from birth and during newborn resuscitation and to increase the value of collected data. The overall objective is to develop an AI system that uses (sensitive) video input to automatically generate timelines of events and activities, starting with time of birth (ToB) and continuing throughout newborn stabilization and resuscitation. Those generated timelines do not themselves contain sensitive information and could be used for documentation, input for clinical debriefing and research on guidelines compliance and treatment effectiveness when combined with outcome data.

In this work, we describe a semi-automated data collection system based on digitally stored informed consent of participants (mothers) and an opt-out option for healthcare providers. Our solution did not interfere with clinical practice and adds minimal burden to healthcare providers. We also describe the data set generated during the study.

## Materials and methods

### Ethics statement

The study has been approved by the Regional Ethical Committee, region west, Norway (REK-Vest), REK number: 222455. Written consent has been obtained from all mothers participating. Healthcare providers were not defined as study participants, and no consent was obtained. The study is registered in the ISRCTN registry: ISRCTN12236970.

### Stavanger university hospital

**Fig 1:**
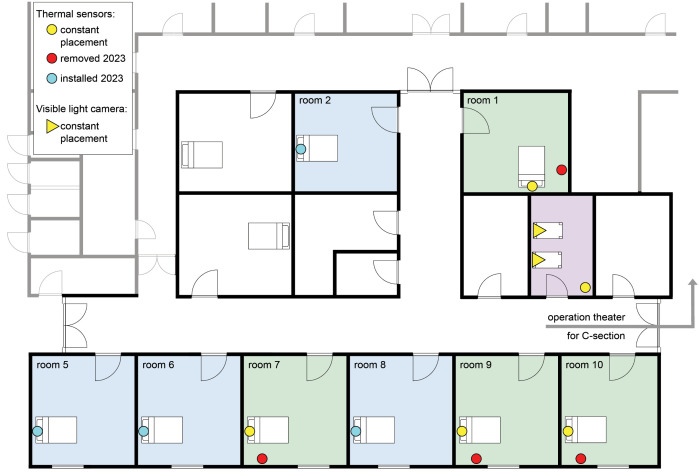
Floor map of the labor department at SUH. Initially, four labor rooms were equipped with two thermal sensors each (green rooms, yellow and red circle indicating placement of sensors). After May 2023, the thermal sensors in red were moved to four additional labor rooms (marked in blue). The pink room is the treatment room, equipped with a thermal sensor and two visible light cameras (yellow triangles) mounted on the treatment tables. Operation theater (two thermal sensors) and anteroom (one visible light camera and one thermal sensor) are not shown on the floor map. *Adapted from Bravida Fire & Security Stavanger with permission.*

All data was collected at the labor ward at SUH. The hospital has ten labor rooms at the labor ward (Fig 1) and one operation theater used for C-sections situated on the same floor. There are four additional labor rooms for low-risk labor on a different floor, that were not included for video collection in this project. Approximately 4000 newborns are born each year at SUH, about 7% need help to start breathing, and 3–4% receive positive pressure ventilation (PPV) [13]. Newborns receiving treatment right after birth that exceeds drying, are placed on a resuscitation station in a treatment room close to, but outside, the labor rooms. Newborns born by C-section are treated at a resuscitation station in the anteroom in conjunction with the operation theater.

### Participants

All women who gave birth at SUH during the study period and had consented were eligible for participation. Pregnant women were informed about and invited to participate in the study during their antenatal visits around pregnancy week 12 and/or 20.

Information about the study and sign-up procedure was provided via continuously playing an information video on screens, as well as in posters and brochures available in waiting areas for the antenatal care at SUH. Starting May 2023 until March 2025, a research assistant or a nurse assistant was available approximately every other day to explain the study to pregnant women and facilitated signing the consent form, either on paper or via a web form. Paper consents were subsequently entered into the digital consent system by a research assistant. Additionally, if the midwives had the capacity, pregnant women were asked to participate upon arrival at the labor ward.

### Data collection

Data collection started November 15^th^, 2021. After complains from medical doctors, supposing that video recordings were illegal surveillance at the workplace, the hospital management paused the data collection on December 7^th^ 2021, but pregnant women were still asked for consent. The management later concluded that the project had all required approvals. May 23^rd^, 2022 data collection resumed and continued until June 30^th^, 2025. When data collection resumed, the project also implemented an anonymous opt-out solution for employees, replacing employee opt-out by email to the PI.

**Fig 2:**
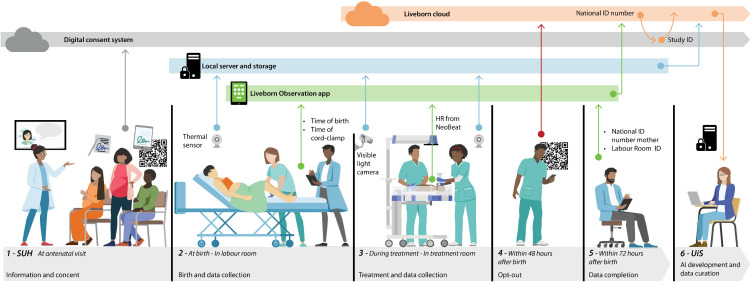
Illustration of data collection components. Data collection started with asking pregnant women for consent (1). During labor thermal and visible light data from labor (2) and treatment (3) were stored locally based on automated triggers. (5) Additional information was entered in the Liveborn observation app and employees could ask for deletion of the data on a web page (4). Eventually data became available for researchers (6).

Fig 2 provides a timeline overview of the data collection components requiring user involvement. Data collection began with informing mothers and obtaining consent during antenatal visits around week 12 and/or 20 of pregnancy. When consent was given via the web interface, this data was sent directly to the digital consent system. If consent was provided on paper, a research assistant would manually input this information into the digital system. The consent covered both collecting thermal videos in the labor room and, if the newborn received treatment, visible light video from the resuscitation station.

At birth, thermal videos from the labor room (or operation theatre) were automatically recorded and stored locally on a server at SUH. A nurse applied NeoBeat newborn heartrate meter (Laerdal Medical AS, Stavanger) on the baby’s chest and documented ToB and time of cord clamp (ToCC) in the Liveborn Observation app. NeoBeat heartrate data was continuously streamed to the app via Bluetooth Low Energy. If the newborn required treatment, visible light video of the treatment area and thermal video from a sensor monitoring the treatment room were automatically captured and stored locally.

Employees could anonymously submit an opt-out request within 48 hours post-birth, which was directly stored in Liveborn cloud. Typically, the day after birth, a research assistant would input the mother’s national ID number and the room where the baby had been born to the respective dataset in the Liveborn Observation app. Following this, the dataset was transferred to the Liveborn Cloud, which verified the consent status associated with the mother’s national ID and retrieved a Study ID from the consent system if consent was confirmed. The mother’s national ID has been deleted immediately after this process, regardless of the consent status.

Local storage contained data from thermal sensors and visible light cameras. Data from local storage (blue horizontal line in Fig 2) was only transferred to the cloud storage if the following criteria were met; maternal consent is available, 48 hours have passed since ToB, and no employee requested to opt-out. The complete dataset was available for researchers to download in Liveborn Cloud no earlier than 48 hours after ToB.

Development of AI algorithms for activity recognition in visible light videos and for detecting birth in thermal videos has been conducted by the research team using UiS infrastructure. Some results based on this dataset have already been published [14–17]. Methods for exploring activity timelines from newborn resuscitation have also been investigated [18].

### Data processing and transfer

The local server ran a data upload script every 6 hours. The input to this script was a queue message received from Liveborn cloud containing ToB, labor room ID, Study ID and the ID of the observation created by the Liveborn Observation app for all new registered births. The detailed steps to collect data from all available sources and combine them to one dataset uploaded to the cloud are described in Table 1. At this point it is important to mention that “thermal videos” were not stored and processed in video format, but in single frame binary files and were consequently processed differently from visible light videos stored in a video formatted file.

**Table 1:** Data processing and transfer to cloud.

Steps performed by data transfer script	Explanation
Check if maternal consent is active	The Study ID is sent to Liveborn cloud, which checks bitUnitor’s consent API and delivers the consent status back to the local server. If consent status is “no”, this information is entered in the database and all further processing is stopped.
Check if at least 48 hours have passed since ToB	No data is processed before the employees had time to send an opt-out request. The time since ToB is calculated, and if less than 48 hours have passed, the birth event is put back to the message queue and will be processed again, next time the script runs.
Check if an employee opt-out request matches with this ToB.	If ToB specified in the opt-out request is within ±15 minutes of the ToB in the birth being processed, the dataset is updated with a “employee opt-out” flag and all further processing is stopped. Opt-out requests are handled independently for labor and treatment data.
The following processing steps are carried out only if none of the previous steps did end the data processing.	
Prepare and upload thermal labor video (if available)	Retrieve all thermal data files that were recorded: by the thermal sensor in the specified labor room AND within ±15 minutes of the registered ToB Combine the files in a zip file and upload them to cloud storage. Filename includes Study ID.
Upload visible light video (if available)	Retrieve all video files that were recorded in the respective treatment room where the video ended after ToB AND started before OR within 10 minutes after the ToB. Convert video files from MKV to MP4 format, disable audio and upload them to the cloud storage. Filename includes Study ID and milliseconds from ToB to video start (can be a negative value if video starts before ToB).
Upload thermal treatment data (if available)	Retrieve all files that were recorded: by the thermal sensor in the same room as the visible light video recording AND within the same time period (start/end of visible light video) Combine the files in a zip file and upload them to cloud storage. Filename includes Study ID.
Update database	Database in cloud storage is updated with data collection status.

The final dataset for each newborn always included Study ID and ToB and either thermal labor data or a visible light treatment video (or both) - depending on if the labor had been in a room with thermal camera and if the newborn received treatment. Additionally, some datasets included ToCC, newborn heart rate data, and thermal video from the treatment room. If consent had been given, but the newborn was born in a room without a camera and did receive resuscitation, no data related to that birth would have been registered, and it would not be part of the database.

Files older than 72 hours were deleted from the local storage, independent of whether data had been uploaded to cloud storage or not. If information from the Liveborn App was uploaded late and not reaching the queue processing within 72 hours after birth, the locally stored data was lost.

If a mother withdrew her consent after data had been collected, the digital consent system informed the data processor and the data for this mother was deleted manually.

### Digital self-service consent

The digital consent system was developed by bitUnitor AS, Norway, based on blockchain using Hyperledger technology hosted on a cloud service provider [19]. It ensured the secure storage and encryption of personal information for participating mothers, and it created Study IDs if consent and data were present. To ensure immutability, the consent status was stored within a blockchain. The digital consent system provided a protected API that enabled the retrieval of consent status and Study ID by authorized applications. The cloud service provided robust security measures for APIs, including centralized access control, encryption, IP address restrictions, and continuous threat monitoring. Data was encrypted both in transit and at rest, ensuring protection against interception and unauthorized access. Additionally, IP address restrictions and virtual network integration were put in place to narrow access to trusted sources only and to minimize potential attack vectors.

### Thermal (infrared) cameras

Thermal sensors offer several advantages for detecting the ToB in labor rooms. These sensors measure thermal radiation in the infrared spectrum and capture significantly fewer details compared to visible light cameras, thereby enhancing privacy for both healthcare providers and women in labor. At the moment of birth, the newborn is slightly warmer than the normal skin temperature of other people in the room, which can be exploited for detection of ToB [17,20,21]. The detection process involves training Artificial Intelligence (AI) models on thermal data, either frame-by-frame or by incorporating spatiotemporal information. How thermal imaging is working and what is affecting its accuracy in this setup has been described by García-Torres et al [22].

The study utilized 12 thermal sensors Mx-O-SMA-TPR079 connected to Mx-S16B camera modules (both Mobotix AG, Germany) to capture data from the labor and treatment rooms. The sensors provided a frame rate of 8.33 frames per second, with an image size of 336 × 252 pixels, and had a field of view measuring 45° × 35°. The sensors were not equipped with a microphone.

The thermal sensors were linked to camera modules via USB cables, and a subsequent wired connection connected those cameras to a secure local network. A Linux server (Ubunto 20.04) within the same network interacted with the cameras over HTTP using the MXCameraSystem SDK 1.0.2 (Mobotix AG, Germany) software.

The cameras were configured to check the temperature every 5 seconds. If at least one pixel exceeding 30° C was detected, the sensor started collecting data for at least 20 seconds. If the temperature exceeded 30° C again during this time, the recording period was extended, including at least the next 20 seconds. This means that if the temperature remained above 30° C, data collection was continued without any breaks. The temperature trigger level was set to a threshold of 30° C to activate data capture when people were present, while avoiding unnecessary recordings when the room was empty. The threshold was set between a normal room temperature and a healthy body temperature, allowing for slight sensor calibration errors while ensuring data was captured while labor was ongoing.

The thermal sensors provided data framewise. The data was stored in files containing data from 100 frames at a time. Those files were stored on a RAID (Redundant Array of Independent Disks) storage together with metadata detailing information about the camera, including the room where the camera was located, the location of the sensor relative to the bed (“head”/ “side”), and the recording start time. This information was used when processing the data where the raw data for the 30-minute period of interest was divided into one frame per file and uploaded to the permanent cloud infrastructure. This file structure allowed for easy post processing like conversion to color-coded temperature images and videos.

#### Sensor placement before May 2023.

**Fig 3:**
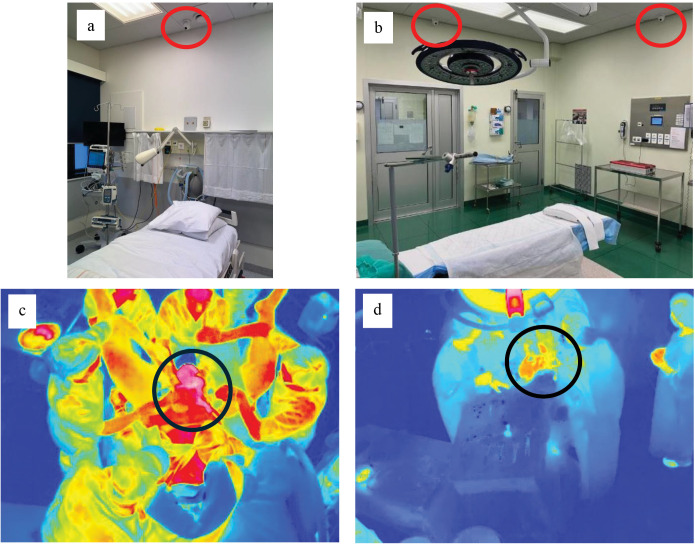
Placement of thermal sensors and example of processed data from ToB. Top: Position of the thermal sensors (marked with a red circle) in a) a labor room, b) the operation theater for C-sections. Bottom: Thermal image at birth showing the complete field of view. c) from a vaginal delivery in the labor room, d) from a C-section delivery in the operating theater. The newborn is marked with a black circle.

In the beginning of the project, four labor rooms were equipped with two thermal sensor modules each (Figs 1, 3). One sensor was positioned on the ceiling directly above and behind the laboring woman’s head (“head-view”), while the other was placed on the right side of the woman in labor (“side-view”). Two thermal sensors were installed on the ceiling in the operating theater for C-sections to obtain a “leg-view” and “side-view” with minimal obstructions in the field of view.

Furthermore, a thermal sensor was strategically positioned on the ceilings of the treatment room and the anteroom, respectively, to monitor the ongoing activities and amount of people present in the room during newborn stabilization or resuscitation (Fig 3c).

#### Sensor placement since May 2023.

In May 2023, the collection of thermal videos was extended to 8 labor rooms. The “side-view” thermal sensors were removed from all labor rooms and installed as “head-view” sensors in four labor rooms that were previously not equipped with thermal sensors (Fig 1).

During the analysis of the thermal videos collected in the initial study period, it was noted that the moment of birth was visible in most cases from the “head-view” data. In instances where it was not visible from the “head-view,” it was neither visible from the “side-view.” Based on these findings, the decision was made to relocate the “side-view” sensor to labor rooms that were not yet equipped with any sensor, to increase the number of recorded births. No modifications had been made to the thermal sensors in the operating theater, treatment room and anteroom.

Fig 3 shows the sensor placement in the labor room and operational theater, respectively and two examples of a thermal image from actual sensors at the ToB.

### Visible light cameras

Visible light videos are suitable to capture stabilization and resuscitation activities, such as stimulation and PPV. Identifying those activities can be done manually, which we did as a part of this research project. However, one of the primary goals of the NewbornTime project was to develop AI models to identify such activities [14,16].

The study utilized three Axis M1134 Network Cameras (Axis Communications AB, Sweden). These cameras operated at a frame rate of 25 frames per second and had a resolution of 1024 × 768 pixels, providing a field of view measuring 90° × 49°.

**Fig 4:**
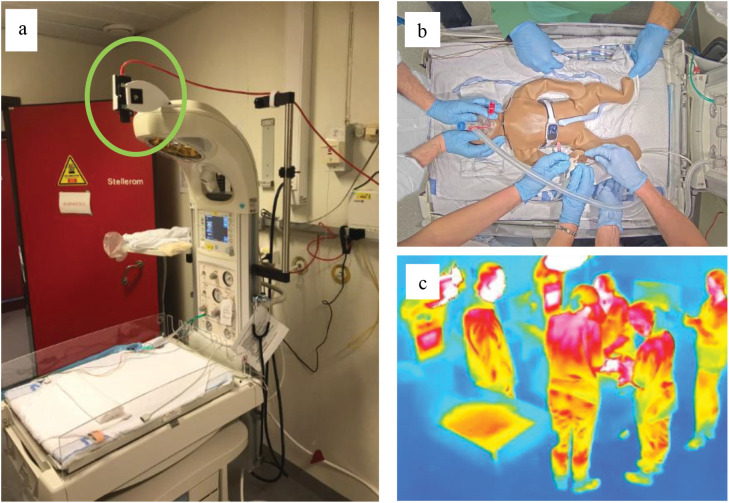
Treatment room equipment and example data. The visible light camera (green circle) was mounted directly above the treatment area **(a)**. Treatment of newborns were captured well with the limited field of view (picture shows simulation using a manikin) (b) and the thermal sensor captured what was happening around the treatment area **(c)**.

The camera’s field of view was adjusted to encompass the smallest possible area of the resuscitation station, ensuring that all treatment actions are captured, while respecting privacy of clinical staff. The camera placement of the visible light camera and an image example from a simulated treatment are shown in Fig 4.

Videos captured by these cameras were streamed directly to local RAID storage. Recording automatically started upon motion detection in the camera’s field of view and continued for 5 minutes after the last detected motion. Each recording also included 60 seconds of footage prior to the first motion trigger. Sound was removed as part of the post-processing steps on the local server at SUH. The motion detection used video processing with a threshold of over 5% frame change for at least 1 second. Video recording started with minimal movement and was often triggered not only by treatment of a newborn, but also by any nearby movement, such as staff cleaning the area, preparing for the next patient or conducting routine equipment checks leading to more footage than just specific patient-related events. All thermal cameras and visible light cameras were time synchronized by using an NTP server hosted on the local network gateway.

#### Camera placement.

All three resuscitation stations in use at the time were equipped with top-down visible light cameras, enabling recording of the surface area of the infant bed (Fig 4a, treatment room in Fig 1). This configuration ensured an unimpeded view of the newborn while minimizing the capture of personal characteristics of the clinicians, such as faces or ID cards (Fig 4b) [14,16].

### Liveborn observation app

**Fig 5:**
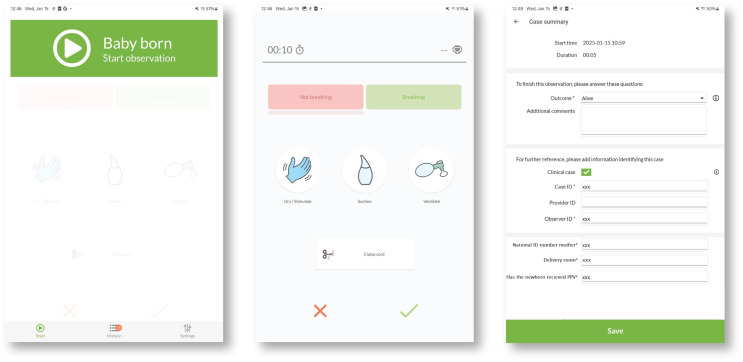
Screenshots of the Liveborn Observation app. Left: start screen where “Baby born” button is pressed to record the ToB. Middle: Event registration screen, that is displayed as soon as “baby born” has been pressed, with “Clamp cord” button, right: Example of summary screen, where research assistant added mothers national ID number and labor room ID.

The Liveborn Observation App [23] (Fig 5), developed by Laerdal Medical AS, Norway, was installed on three Android tablets (Samsung Galaxy Tab A7 Lite) that were placed in the labor ward. Once a birth was expected, a midwife assistant brought a tablet to the labor room. In real-time, the midwife assistant annotated ToB and ToCC by pressing the respective on-screen buttons in the app. The app received newborn heart rate data from NeoBeat continuously while active.

A research assistant added additional case information to the app, such as the labor room and the personal national ID number of the mother.

When data collection for the NewbornTime project started, there was already an ongoing study called NeoBeat efficacy study [24] that recorded video of newborn stabilization and resuscitation. To streamline our efforts, we opted to utilize the existing infrastructure and incorporate a digital consent processing and thermal video processing step. The registration of ToB by an in-app button at birth was initially introduced by the NeoBeat efficacy study at SUH. We utilize the information this provided to determine the start and end timestamps for inclusion of the thermal video. Kolstad et al. [25] showed that the button was pressed with a median deviation of less than 2 seconds compared to ToB annotation from manually studying thermal videos.

### Employees opt-out

Employees were not defined as study objects but had the option to request deletion of videos from labors or resuscitations where they had participated, within 48 hours after the respective ToB. This could be done anonymously through a web interface accessible by scanning a QR code provided in each labor and treatment room or entering the webpage directly. The employee needed to specify the date, time and room of the capture of the video that was requested to be deleted and confirm that he or she was present.

### Infrastructure for data processing and storage

**Fig 6:**
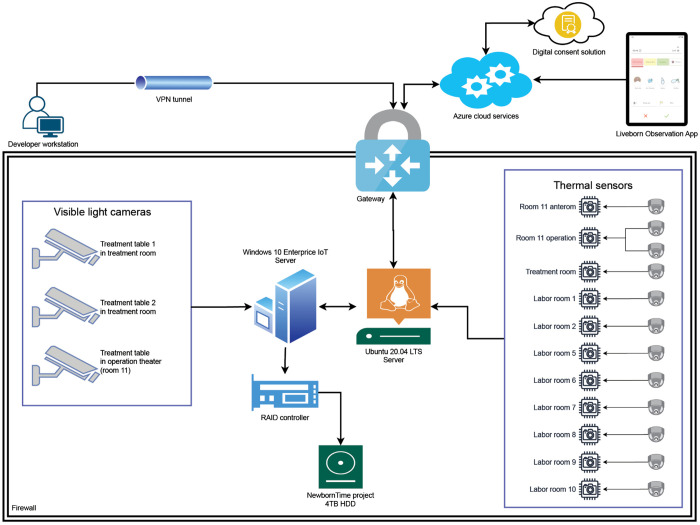
Data collection infrastructure at SUH. The local infrastructure at SUH consisted of one Windows server responsible for hosting the RAID storage and one Ubuntu server responsible for collecting data from the thermal sensors, processing the dataset and uploading data to the cloud. All devices were communicating on a closed network separated from the hospital network. Developers had access to the network using a VPN tunnel for continuous development and monitoring of the system.

Fig 6 shows an overview of the data collection infrastructure at SUH for the NewbornTime project.

The project used 12 thermal sensors and 3 visible light cameras, along with 2 servers. One server was a Windows 10 Enterprise server, while the other was an Ubuntu 20.04 LTS server. The Windows server had been set up for an earlier project and was reused for hosting the data storage unit, which included a RAID level 5 controller with 8 TB of effective storage, where 4 TB were allocated specifically to this project. The Linux server ran the software developed for the project. All software hosted on this server ran as containerized Docker applications, handling data collection from the thermal sensors as well as data processing and deletion processes. The data captured by the visible light cameras was directly stored on the RAID.

All IoT devices and servers were communicating on a closed network, physically separated from existing IT infrastructure at SUH. This means that access to this network and all resources within it was managed by the project. All communication going in and out of the network was controlled by a gateway where a selected few could access the system via VPN tunnels on a need-to-know basis. Communication with Azure cloud services was managed by a site-to-site VPN connection, and a DNS server was implemented in the Azure services to resolve domain names.

The cloud infrastructure consisted of multiple virtual networks and subnetworks allocated to different resources. This means that the data storage resources (database, blob storage) were segregated from the rest of the infrastructure. Connections between various resources within Azure were established using private links.

Researchers at UiS could access video data stored on the blob storage through a second site-to-site VPN communication channel established between the blob storage subnetwork and the local secured server at the university. This data transfer was essential to ensure that the data was accessible for computationally intensive AI algorithm development in a secure environment.

The infrastructure comprised three public-facing web applications: the digital consent system, the Liveborn system, which is responsible for collecting metadata information regarding births from the Liveborn Observation app, and the web application that enables employees to submit opt-out requests.

## Results

### Consents

Between November 15^th^, 2021, and June 30^th^, 2025, 3852 women consented to participate in the study. 3723 allowed their data to be used for educational purposes as well. The average daily consent rate was 0.6 until May 2023. This rate increased to 5.0 per day during the period when nurses and research assistants actively informed participants about the study. The inclusion rate declined again to 0.3 per day after March 2025, when this direct approach was discontinued. From May 2023 onward, the consent rate among women personally approached was approximately 90%, consistent with rates reported in video-based quality improvement studies [2].

**Fig 7:**
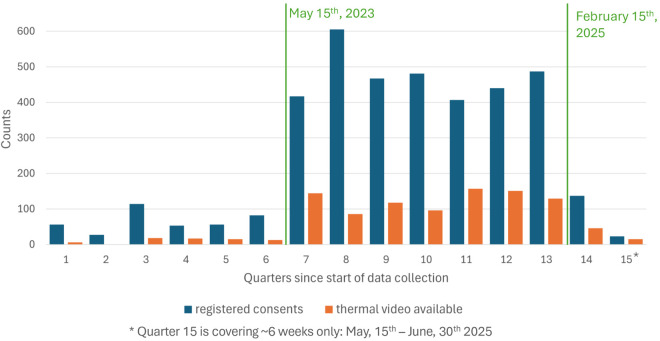
Histogram showing number of maternal consents registered and thermal labor videos collected per quarter. The rise in consents during quarters 7–13 coincides with research assistants actively approaching mothers. The increase in collected thermal videos is further explained by expanding data collection to eight labor rooms.

Fig 7 shows the number of thermal videos available in quarters with a significant increase in available data from Quarter 7 (May 2023), when more consents were collected and the amount of labor rooms equipped with thermal sensors had doubled from 4 to 8 rooms. Data collection was on hold in complete quarter 2 and partly quarter 1.

### Collected data – consort diagram

**Fig 8:**
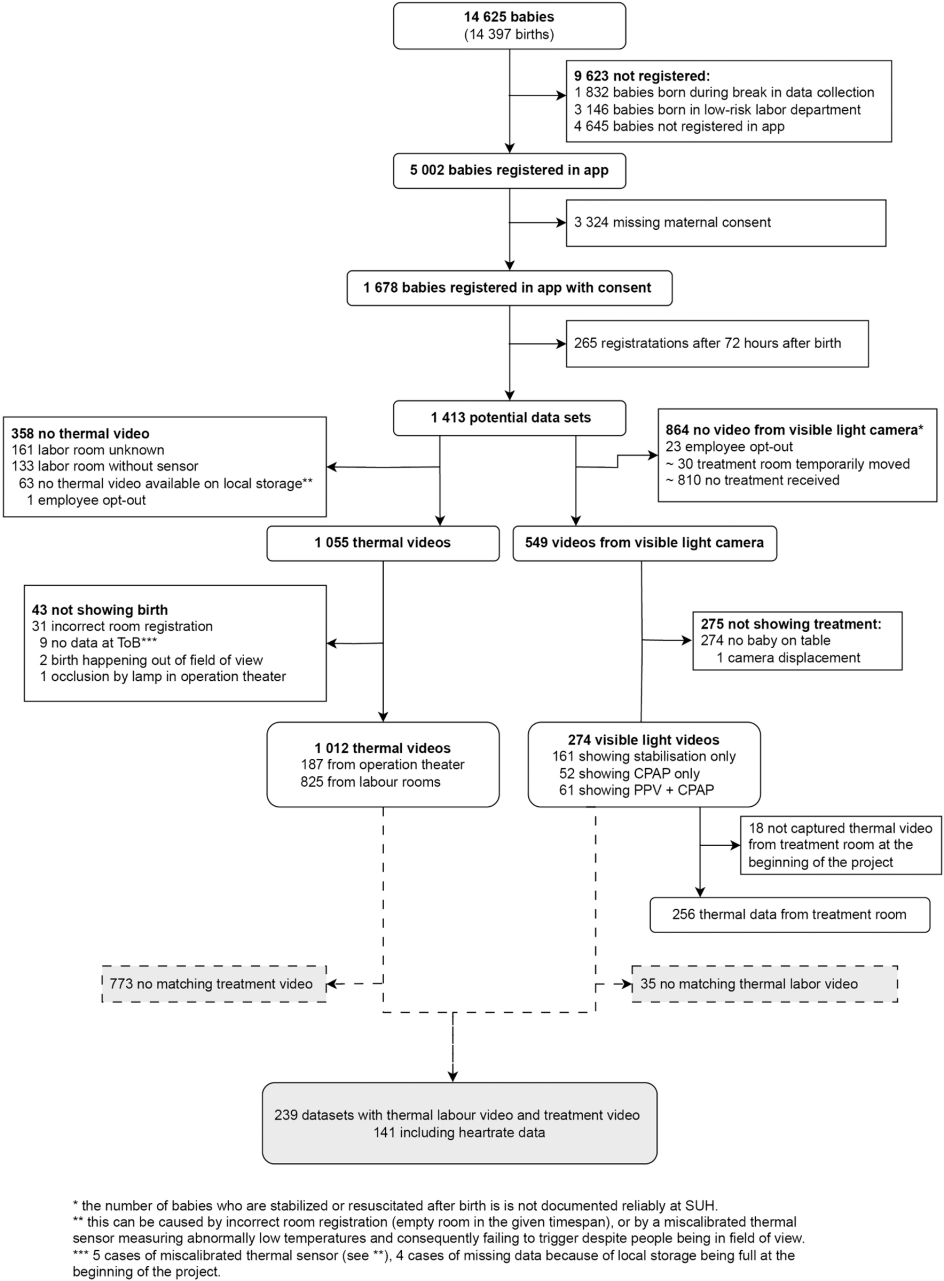
Consort diagram. Flow chart showing the amount of collected data and reasons for dropout.

During the reported period, 14.397 births took place at SUH, resulting in 14.625 babies being born. The project collected 1012 thermal videos from labor showing birth and 274 treatment videos showing newborn stabilization or resuscitation. Those datasets overlap in 239 records, whereof 141 records have continuous newborn heartrate data available. The detailed consort diagram is shown in Fig 8.

9623 babies were not included in the study. About half due to being born in the low-risk labor ward or during the pause in data collection, while the other half were never registered in the app. Additionally, 3324 babies were excluded due to missing maternal consent. Potential data from 265 births/newborns was lost due to late submission of the app data (video data was automatically deleted before the system knew about birth).

Out of the collected visible light videos, 275visible light did not show a baby. This is due to the data collection setup, which was designed to accept capturing some videos showing an empty treatment area, in order to avoid missing any that show treatment of a newborn.

The 274 visible light videos showing treatment included 52 videos showing CPAP and additional 61 videos showing CPAP and PPV treatment. As there was no reliable data available on the number of newborns receiving treatment after birth, we do not know how often the motion trigger failed. However, the big amount of visible light videos not showing a baby is an indication of a low failure rate of the motion trigger.

Duration of videos of newborn stabilization and resuscitation ranged from 8 to 60 minutes, with 60 minutes being the pre-defined maximum recording length. The videos have not been annotated to indicate the duration of time the newborn is on the table or receiving treatment. A longer video does not necessarily mean a longer treatment episode, as it may also include extended periods of an empty treatment area.

There was no thermal video available from 358 births; the main reason is unknown labor room or labor room without thermal sensor. 63 births were lacking thermal data on local storage. This was caused by incorrect room numbers pointing to labor rooms that were not in use at registered ToB or miscalibrated sensors measuring abnormally low temperatures, failing to trigger data collection despite people being present. To conservatively estimate the failure rate of the temperature trigger, we could assume all 63 videos being lost due to trigger failure. That would suggest a thermal trigger failure rate of 4.5%.

Unregular self-calibration of the sensor lead to some missing frames in some videos. In nine videos there were multiple frames missing leading to a thermal video not showing the actual ToB, four of those happened during a storage issue in the beginning of the project, five of those were due to a miscalibrated thermal sensor, which reported abnormally low temperatures and failed to trigger around ToB, but triggered when hot objects like a bowl of hot water were present in the field of view.

Each thermal video showing labor covered a 30 minute period with registered ToB placed in the middle of the dataset. From 1012 collected thermal videos, 825 show birth in a labor room while 187 were recorded during a c-section in the operation theater.

Fig 7 shows the number of available labor videos over time compared to the collected consents in the same time period. 3558 pregnant women gave consent in 2025, most of them were still pregnant at the time data collection for this study ended. We therefore did not count them in the following calculation. We collected thermal and/or visible light videos from 1012 individual newborns, resulting in only 29% of the registered maternal consents leading to data inclusion.

### Employees opt-out requests

After introduction of the anonymous employee opt-out web solution in May 2022, 98 requests were registered: 3 for thermal labor videos and 95 for visible light videos. Of those, 23 requests resulted in the deletion of a visible light video and 1 request deleted a thermal video from labor. The remaining 74 requests concerned datasets already unavailable due to no registered matching birth, missing maternal consent or missing local data.

**Fig 9:**
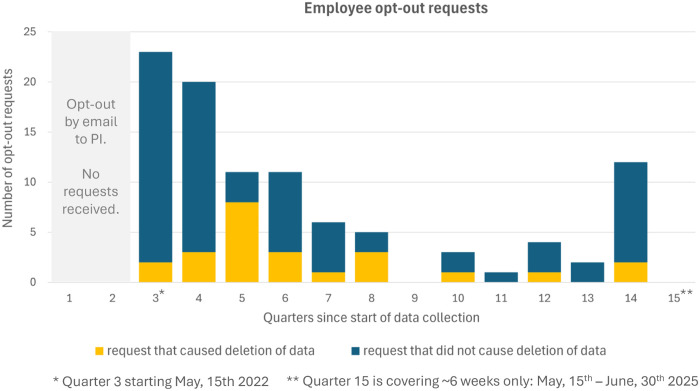
Amount of employee opt-out requests over time.

In the first quarters of data collection, no employee asked for deletion by email to the PI. After implementation of the anonymous opt-out web interface in May 2022, 23 opt-out requests were recorded in the first quarter, decreasing constantly to less than 5 requests per quarter and kept low, but increased to 12 requests in quarter 14 (Fig 9). There were no opt-out requests registered after May 1^st^, 2025.

While not formally studied, the initial decrease in opt-out requests may be due to individuals becoming accustomed to being filmed. The increase in requests in spring 2025 coincides with discussions about a follow-up project at SUH, likely to raise awareness about the ongoing data collection.

## Discussion

### Consent

In their study, Gelbart et al. [26] examined the ethical and legal aspects of video recording during newborn resuscitation. They highlighted the challenge of obtaining consent during antenatal care, as it could potentially cause unnecessary anxiety for parents. On the other hand, obtaining consent during or after labor may not provide a fully voluntary choice due to the emotional state of the parents at that time.

In the NewbornTime project, the regional ethical committee required antenatal consent from the pregnant women. While the project was enthusiastic about a digital, self-serviced consent system designed to be user-friendly and secured by blockchain technology, we learned that the pregnant women rarely used the opportunity to sign up proactively although study information was presented in brochures and videos in the waiting areas for antenatal controls. While we experience high percentage of consent for pregnant women who were approached by a project member, the project did not have enough resources to cover personnel costs to ask all expecting mothers for consent.

Missing maternal consent was a major reason for data exclusion in our study. Simultaneously we only collected data for 29% of the registered consents. To address this, we need to improve the consent process in future projects and invest resources to better inform potential participants, aiming for a greater overlap between consents given and data collected.

### Challenges of semi-automated data collection

We experienced data loss when manual inputs were forgotten or not registered timely, especially in holiday seasons with decreased availability of qualified personnel and a higher workload of the remaining staff. In future projects, if the ToB detection algorithm is implemented, manual interaction at birth will be unnecessary and data collection automated to cover all births with consent. The thermal video being lost due to wrong or missing information about where the labor happened could be solved technically, for example by registering the mother’s national ID number on a device in the room that could forward that information to the Liveborn cloud or local storage.

The big amount of visible light videos not showing a newborn would be easy to avoid if reliable data on treatment of each newborn would be easily available within days after birth. A labor ward setup where the treatment tables are placed in each individual labor room would help additionally in collecting continuous and complete datasets from each newborn.

### Future perspectives

While video review and annotation are time consuming activities, future AI generated timelines of birth and resuscitation activities would arise with little effort. Automatization also allows for scaling up data collection efforts and increasing the amount of data, which can lead to a better understanding of the effect of time-critical activities around birth.

Generated timelines can easily be anonymized and can be incorporated in structured documentation of resuscitation events, data-guided debriefing systems, quality improvement systems, and retrospective analysis for evidence-based research around newborn resuscitation guidelines and efficiency of treatment.

For AI development, we manually annotate ToB, ToCC, ventilation (CPAP, PPV), stimulation and suction. We also work on semi-supervised algorithms to support the time-consuming annotation work. This may also help to adapt the AI models for ToB detection and timeline generation to new hospitals with different environments.

We believe that the integration of thermal imaging and AI-based activity recognition from video can be extended to other non-diagnostic healthcare applications, such as documenting activities in emergency care or security surveillance, while maintaining privacy for both healthcare providers and patients.

Our group recently published results from AI models developed to detect ToB from thermal videos of labor [17]. In our evaluation, the model successfully identified a ToB in all 35 test cases, achieving a median error of 2 seconds and a mean error of 4.5 seconds. The model was originally trained on approximately 600 videos, and with a dataset that has since increased by about 50%, we are now positioned to retrain it.

Once video recording becomes standard care, the need for patient consent would cease, enabling a fully automated data collection system. In the meantime, there might be a bigger acceptance of using video as input to AI algorithms, generating a timeline of the newborn’s treatment. AI algorithms could detect ToB from data streamed from thermal sensors and generate timelines from the visible light treatment video. The only manual input required would be a patient identifier for proper data storage. Using videos exclusively to automatically create precise timelines could effectively address privacy concerns while still preserving essential information about the patient’s treatment.

## Conclusion

This work describes the data collection system for recording thermal videos from the labor room and time synchronized visible light videos from newborn stabilization or resuscitation. The system has successfully recorded over 1000 videos from labor and newborn resuscitations. Encountered challenges and suggestions on how to solve them to make the data collection system scalable are discussed.
